# When wanting to change is not enough: automatic appetitive processes moderate the effects of a brief alcohol intervention in hazardous-drinking college students

**DOI:** 10.1186/1940-0640-7-25

**Published:** 2012-12-07

**Authors:** Brian D Ostafin, Tibor P Palfai

**Affiliations:** 1 Department of Psychology, University of Groningen, Grote Kruisstraat 2/1, 9712, TS, Groningen, The Netherlands; 2 Department of Psychology, Boston University, Boston, MA, USA

**Keywords:** Automatic processes, Alcohol, Implicit association test, Motivational intervention, Self-control, Self-regulation, Addiction

## Abstract

**Background:**

Research indicates that brief motivational interventions are efficacious treatments for hazardous drinking. Little is known, however, about the psychological processes that may moderate intervention success. Based on growing evidence that drinking behavior may be influenced by automatic (nonvolitional) mental processes, the current study examined whether automatic alcohol-approach associations moderated the effect of a brief motivational intervention. Specifically, we examined whether the efficacy of a single-session intervention designed to increase motivation to reduce alcohol consumption would be moderated by the strength of participants’ automatic alcohol-approach associations.

**Methods:**

Eighty-seven undergraduate hazardous drinkers participated for course credit. Participants completed an Implicit Association Test to measure automatic alcohol-approach associations, a baseline measure of readiness to change drinking behavior, and measures of alcohol involvement. Participants were then randomly assigned to either a brief (15-minute) motivational intervention or a control condition. Participants completed a measure of readiness to change drinking at the end of the first session and returned for a follow-up session six weeks later in which they reported on their drinking over the previous month.

**Results:**

Compared with the control group, those in the intervention condition showed higher readiness to change drinking at the end of the baseline session but did not show decreased drinking quantity at follow-up. Automatic alcohol-approach associations moderated the effects of the intervention on change in drinking quantity. Among participants in the intervention group, those with weak automatic alcohol-approach associations showed greater reductions in the amount of alcohol consumed per occasion at follow-up compared with those with strong automatic alcohol-approach associations. Automatic appetitive associations with alcohol were not related with change in amount of alcohol consumed per occasion in control participants. Furthermore, among participants who showed higher readiness to change, those who exhibited weaker alcohol-approach associations showed greater reductions in drinking quantity compared with those who exhibited stronger alcohol-approach associations.

**Conclusions:**

The results support the idea that automatic mental processes may moderate the influence of brief motivational interventions on quantity of alcohol consumed per drinking occasion. The findings suggest that intervention efficacy may be improved by utilizing implicit measures to identify those who may be responsive to brief interventions and by developing intervention elements to address the influence of automatic processes on drinking behavior.

## Background

Young adults who engage in heavy alcohol consumption are more likely to experience both current and future alcohol-related problems. Heavy episodic drinking is associated with risky behaviors, such as having multiple sex partners [1] and a variety of negative academic, physical, and social consequences [2,3]. Additionally, these drinking patterns predict concurrent and future alcohol use disorders [4]. Brief interventions may lead to reductions in the drinking behavior of young adults, but these reductions appear to be modest [5]. Examining variables that moderate intervention success may help to predict who will benefit from treatment [6]. One potential class of moderators consists of mental processes that are automatic—motivational responses that are spontaneously activated in the presence of an alcohol cue (in contrast to deliberative consideration of whether to consume). The current study examined whether automatic alcohol-approach associations would act as a moderator of a brief alcohol intervention.

Brief motivational interventions (BMIs) represent a class of treatments designed to reduce heavy drinking, in part through increasing motivation and commitment to change. Although there are differences between specific BMIs [7], they typically involve one or two sessions and consist of elements such as individualized feedback of drinking behavior and negative alcohol-related consequences, emphasis on the responsibility of the individual to make any change, offering advice to change, offering options for making a change, enhancing the individual’s self-efficacy for change, and conducting the intervention in an empathic style [8]. In BMIs, motivation to reduce drinking behavior is elicited through a number of strategies such as discussing discrepancies between the individual’s values (e.g., academic performance) and actual behavior (e.g., frequently neglecting homework because of drinking alcohol). A key objective is to enhance motivation and commitment to change by increasing the salience of negative alcohol-related consequences and highlighting their incongruence with personal standards and valued outcomes.

Early research indicated that BMIs may be a useful treatment for hazardous drinking among college students [9-12]. Recent reviews suggest that BMIs for alcohol and other substance use yield beneficial effects [13], but that these effects are modest in size [5]. In an effort to identify those who may benefit from motivational interventions for alcohol use, investigators have begun to explore the role of a number of moderators [6]. Regarding participant variables, there is mixed evidence for gender as a moderator of BMIs, with some studies supporting it [14] and others finding nonsignificant results [9,15]. Other potential moderators, such as self-regulation skills and tendency to engage in social comparison, have failed to predict who will benefit from BMIs [15].

There is growing support for the view that alcohol use may be influenced by two systems of psychological processes: (1) an automatic, non-volitional, impulsive system, and (2) a resource dependent, volitional, reflective system [16-18]. This model suggests that, for heavy drinkers, the typical response to an alcohol cue is an automatically activated disposition to approach and consume and that sufficient motivation to restrain and sufficient self-control resources are required to inhibit this automatic appetitive response [19]. From this perspective, although BMIs may elicit motivation to change drinking, strong automatic alcohol-approach associations may impede the ability to translate this motivation into actual change. Thus, one class of moderators that may be particularly important to explore consists of implicit measures of automatic approach responses to alcohol. In contrast to explicit measures, which require participants to directly introspect and report on mental content (e.g., outcome expectancies), implicit measures are designed to assess automatic processes related to mental content by using indirect methods such as reaction time tasks [20]. A number of implicit measures have been used to assess the role of automatic attentional biases and mental associations in substance use behavior. For example, attentional biases toward alcohol cues are related to heavier alcohol consumption [21-23] and have been shown to predict treatment success in individuals motivated to abstain or restrain alcohol [24] and other drug use [25,26].

One widely-used implicit measure of automatic affective associations with target stimuli (such as alcohol) is the Implicit Association Test (IAT) [27]. The IAT is a categorization task that allows inferences about the relative associative strength between two concepts through reaction time performance (see the Methods section for more procedural detail on the IAT). Research with young adult drinkers indicates that automatic alcohol-affect associations measured by the IAT demonstrate a medium effect-size relation with alcohol use, even when controlling for explicit measures of alcohol-affect associations [28-30]. Of particular importance for clinical research, the IAT has been shown to predict failure to control alcohol consumption [31,32].

Theoretical accounts suggest that automatic mental processes can impede the ability to change addictive behaviors [16]. The research reviewed above supports this theory by providing evidence that the strength of automatic appetitive responses (i.e., attentional biases [24] and approach associations [31,32]) to alcohol cues predict alcohol consumption despite motivation to restrain use. It follows that the effects of increasing motivation to change drinking behavior with BMIs may be moderated by automatic processes. Specifically, strong automatic alcohol-approach associations may impede the ability to translate motivation to change drinking into actual reductions of alcohol consumption.

The current study was designed to examine whether a measure of automatic alcohol-approach (relative to alcohol-avoid) associations would moderate the efficacy of a BMI in a sample of hazardous drinkers. It was hypothesized that automatic alcohol-approach associations would moderate intervention effects such that students exposed to a BMI would show greater reductions in amount of alcohol consumed per occasion if they had weaker automatic alcohol-approach (relative to alcohol-avoid) associations. We also examined whether automatic alcohol-approach associations would moderate the relation between individual differences in motivation to change drinking and subsequent drinking quantity. We predicted that, among those with high motivation to change, subsequent reductions in consumption would occur in those with weak alcohol-approach associations but not in those with strong alcohol-approach associations.

## Methods

### Participants

Eighty-nine university student hazardous drinkers participated in the study for course credit. The inclusion criterion consisted of reporting hazardous drinking behavior, assessed as a score of 8 or more on the Alcohol Use Disorders Identification Test (AUDIT) [33]. The exclusion criterion consisted of having a language other than English as the native language. One participant did not return for the follow-up session, and one participant was dropped from analyses because English was not her native language.

### Measures

#### Hazardous drinking

Hazardous drinking behavior was assessed with the AUDIT [33], a 10-item measure designed to measure harmful drinking. Each item is scored from 0 to 4, with response options indicating quantity of use (ranging from *1 or 2* to *10 or more*) or frequency of alcohol use or occurrence of alcohol-related problems (e.g., ranging from *Never* to *Daily or almost daily*).

#### Alcohol involvement

Alcohol use at baseline and at follow-up were assessed with questions similar to those proposed by NIAAA [34]. Participants reported their frequency of alcohol use over the past year at baseline by selecting one of 9 options ranging from 0 (*I didn*’*t drink alcohol in the past year*) to 8 (*Every day*) and over the past month at follow-up by selecting 1 of 8 options ranging from 0 (*I didn*’*t drink alcohol in the past month*) to 7 (*Every day*). Participants reported their average amount of alcohol consumed over the past year at baseline and over the past month at follow-up by selecting one of 12 options ranging from 0 (*I didn*’*t drink alcohol in the past year or past month*) to 11 (*13 or more total drinks*).

#### Readiness to change drinking behavior

Motivation to reduce drinking behavior was assessed using a Readiness to Change Ladder adapted from previous work [35]. Subjects circled a single number on a pictorial ladder with rungs from 0 (*No thought of changing*) to 10 (*Taking action to change*) to indicate motivation to change their drinking. This type of measure has been used to assess motivation to change alcohol use in previous research [36,37].

#### Implicit measure of alcohol-approach associations

Automatic approach associations with alcohol were assessed using the IAT [27] and presented on E-Prime software [38]. In the IAT, participants use two response keys to categorize stimuli into four categories: two target categories (i.e., images of beer and water) and two attribute categories (i.e., approach-related words [advance, anticipate, approach, closer, hope] and avoidance-related words [avoid, away, escape, leave, withdraw). In two sets of combination blocks, each response key was paired with both an attribute and a target category (i.e., left key = approach or beer, right key = avoid or water) such that over the two combination block types, each attribute category was paired with both target categories.

The IAT was presented in seven blocks: (1) a 10-trial target discrimination block, in which the beer stimuli were categorized with the left key and water stimuli with the right key (for the congruent-block-first condition); (2) a 10-trial attribute discrimination block (left = approach and right = avoid); (3) an 8-trial combination block (left = beer + approach and right = water + avoid), (4) a 40-trial combination block with the same combination as block 3; (5) a 10-trial discrimination block wherein “beer” and “water” switched positions (for the congruent-block-first condition, left = water and right = beer); (6) an 8-trial combination block (left = water + approach and right = beer + avoid); and (7) a 40-trial combination block with the same combination as block 6. Two IAT orders were used, one with the beer + approach (and water + avoid) combination block first and one with the water + approach (and beer + avoid) combination block first. IAT order was counterbalanced across participants.

IAT scores are calculated as difference scores between the response times in the congruent and incongruent combination blocks (blocks 4 and 7) with larger difference scores indicating stronger automatic approach associations towards alcohol relative to alcohol-avoid associations. The IAT score was calculated with the D_1_ algorithm [39] with the modification that the data from the lead-in trials and the first combination blocks were not used in scoring. Data from these trials were not collected because this study was run before the publication of the new scoring algorithm.

#### Assessment of alcohol-related problems for intervention

Two measures were administered to assess negative consequences related to alcohol that could be used in the brief intervention: (1) the Young Adult Alcohol Problems Screening Test (YAAPST) [40], which is a 36-item expanded measure of the original YAAPST [41] designed to assess alcohol-related problems relevant to college students (e.g., poor grades because of drinking) and the general population (e.g., driving while intoxicated); and (2) the Short Inventory of Problems (SIP) [42], a 15-item measure that assesses alcohol-related problems across a number of domains such as physical health and relationships. Participants reported the frequency of experiencing each of these 51 items over the previous year with response options ranging from “*No*, *never*” to “*40 or more times in the past year*.”

### Procedure

The first author conducted the interventions. At the time of the study, the author was an advanced doctoral student in clinical psychology and had training and clinical experience in administering BMIs. The study was approved by the Boston University Institutional Review Board (IRB). The study consisted of three sessions. An initial session was conducted to screen for hazardous drinking behavior. Participants were tested in groups of 5 to 20 and first completed an IRB-approved informed consent form. The screening questionnaire included the AUDIT as well as a selection of five beer brands, from which participants were to choose one as the most “desirable/positive” (this rating was used to select stimuli for the IAT in the second session). After completing the session, experimenters scheduled each AUDIT-positive student for a second session within a week.

Participants completed the second session individually and were randomly assigned to either an intervention (*n* = 44) or control (*n* = 43) condition. Participants began the session by completing the IAT. After this, participants completed measures of drinking behavior and alcohol-related problems over the previous year as well as the baseline Readiness to Change Ladder (one participant did not complete the baseline Readiness to Change Ladder) and a number of personality measures unrelated to the current study. After finishing this packet, participants took a five-minute break, during which time the experimenter selected 10 alcohol-related problems (from the SIP and YAAPST) that were endorsed as having occurred most frequently over the past year to use for the intervention group. In order to avoid creating excessive discomfort for participants, no sex-related consequences (e.g., having been pressured or forced to have sex when drunk) were selected for the intervention.

After the break, the intervention-group participants took part in a discussion about the alcohol-related problems that they reported as having occurred most frequently over the past year. The experimenter informed the participants that the study was interested in students’ attitudes towards various health behaviors, and that their discussion would be about what they like and dislike about drinking. The experimenter asked for and received verbal consent to begin the discussion. In order to build rapport, participants were first asked the open question of what they liked about drinking. The central component of the intervention was adapted from an earlier study [11] and consisted of the following three questions for each of the 10 selected alcohol problems: (a) “Can you give me an example of that outcome?”; (b) “What is it about that consequence that you do not like?”, and (c) “Does this happen if you are not drinking?”. In order to encourage more thorough processing and discussion of these consequences on the part of the participants, the discussion was conducted in an empathic style (in which the interviewer was nonjudgmental about the participants’ behavior and experience) and utilized reflective listening (reflecting back the meaning of participants’ statements, especially those that communicated acknowledgment or concern about the negative alcohol consequences), as discussed in the motivational interview literature [43]. The discussion lasted until either there were no more alcohol problems to discuss or until 15 minutes had elapsed. After this, intervention participants completed the post-intervention Readiness to Change Ladder. Control participants completed the post-intervention Readiness to Change Ladder immediately after the five-minute break.

At the end of this session, participants were scheduled to return for a follow-up session six weeks later. In the follow-up session, participants reported their drinking behavior over the previous month.

### Statistical methods

The quantity of alcohol consumed per occasion and Readiness to Change Ladder variables were not normally distributed and were consequently log-transformed for data analyses. The main hypothesis that automatic alcohol-approach associations would moderate the relation between the intervention condition (independent variable) and changes in alcohol consumption at follow-up (dependent variable) was examined with a hierarchical regression analysis. We used a regression analysis of change in quantity of alcohol consumed per occasion (follow-up quantity/occasion minus baseline quantity/occasion [both log-transformed]) on the IAT score and group condition entered as Step 1, and a product of the standardized values of the IAT score and group condition entered as Step 2. Group differences were examined with t-tests and ANOVAs. The data were analyzed with the SPSS Windows V.19.0 program.

## Results

### Demographics and drinking variables

The final sample was mostly female (*n* = 54) and White (*n* = 78) with a mean age of 18.5 years (*SD* = 0.7). At baseline, participants reported drinking alcohol an average of 1.5 (*SD* = 1.1) times per week and 5.0 (*SD* = 1.8) drinks per occasion over the previous year, a mean AUDIT score of 12.3 (*SD* = 4.1) a mean Readiness to change score of 2.8 (*SD* = 3.0), and a mean IAT score of −0.8 (*SD* = 1.1). At baseline, the BMI group reported drinking more alcohol per occasion than the control group, *t* (85) = −2.7, *p* = 0.01. The BMI group was not different from the control group in frequency of use (*t* [85] = −0.3, *p* = 0.73), AUDIT score (*t* [85] = −1.1, *p* = 0.29), readiness to change drinking (*t* [85] = −0.4, p = 0.68), or IAT score (*t* [85] = −0.9, *p* = 0.37). At the follow-up session, participants reported drinking 1.7 (*SD* = 1.4) times per week and 5.3 (*SD* = 2.7) drinks per occasion over the previous month. At follow-up, there were no group differences in alcohol consumed per occasion (*t* [85] = −1.6, *p* = 0.13) or frequency of use (*t* [85] = 0.7, *p* = 0.48).

### Intervention effects on readiness to change drinking

We first examined whether the brief intervention would increase motivation to reduce drinking behavior. We examined this by comparing the groups on a difference score of post-intervention Readiness to Change Ladder minus baseline Readiness to Change Ladder (both log-transformed), with larger scores indicating a greater increase in motivation to reduce drinking. The results from an ANOVA indicated that motivation to reduce drinking behavior increased more for the intervention group (*M* = 0.09, *SE* = 0.02) than the control group (*M* = 0.008, *SE* = 0.02), *F* [1, 84] = 7.1, *p* = 0.009).

### Interaction analysis of IAT and intervention

**Figure 1:**
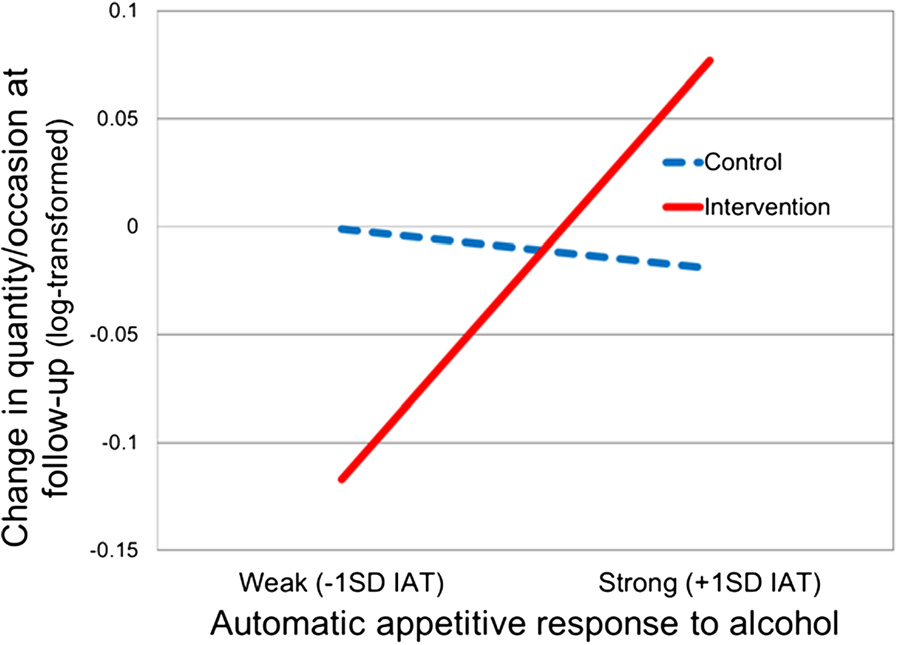
**Automatic appetitive associations with alcohol moderate the effects of a brief motivational intervention on changes in quantity of alcohol consumed**/**occasion at follow**-**up.**

The main hypothesis was that the intervention effects on quantity of alcohol consumed per occasion would be moderated by automatic alcohol-approach associations. The results of the moderator regression analysis (Table 1) indicate an absence of a main effect for the intervention on change in alcohol consumed per occasion (*β* = −.02, *p* = 0.83) and a significant interaction effect (*β* = 0.29, *p* = 0.007). Following Aiken and West [44], the interaction effect was probed to examine whether there were differences between groups at high and low levels of IAT scores. Specifically, we tested whether there was a significant difference between the group regression lines at low (−1 *SD*) and high (+1 *SD*) levels of automatic alcohol-approach associations. The results of these analyses showed that the difference between intervention and control conditions in quantity consumed per occasion was only significant for those with weak automatic alcohol-approach associations on the IAT (−1 *SD*) (*t* = −2.17, *p* = 0.03). The difference between groups for those who exhibited strong automatic alcohol-approach associations on the IAT (+1 *SD*) was not significant (*t* = 1.78, *p* = 0.08). These findings are illustrated in Figure 1.

**Table 1:** **Automatic appetitive responses to alcohol moderate the effect of an intervention on changes in alcohol consumed per occasion** (**N = 87**)

	**Variable**	***R***-***squared change***	***F***-***change***	***Beta***
***Change in quantity of alcohol consumed per occasion between baseline*** &***follow***-***up***				
Step 1				
	IAT Score	0.04	*F* (2, 84) = 1.83	0.21
	Group Condition			−0.02
Step 2				
	IAT x Group Condition	0.08	*F* (1, 83) = 7.62	0.29*

*Note*: Change in quantity of alcohol consumed per occasion = follow-up quantity/occasion – baseline quantity/occasion (larger values indicate increased consumption); Group Condition (0 = Control; 1 = Intervention); IAT score (larger scores = stronger automatic appetitive responses toward alcohol)

*p < 0.05.

### Interaction analysis of IAT and readiness to change drinking

**Figure 2:**
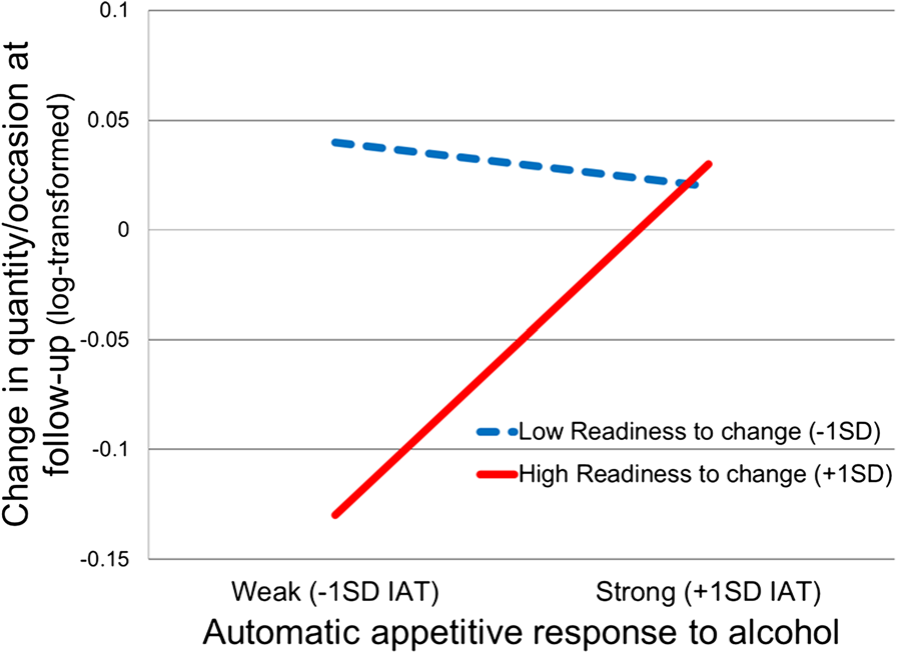
**Automatic appetitive associations with alcohol moderate the relation between motivation to change drinking behavior and changes in quantity of alcohol consumed**/**occasion at follow**-**up.**

In order to further explore the nature of the moderating effects of automatic alcohol-approach associations, we examined the extent to which the IAT score would moderate the relation between motivation to reduce drinking and changes in drinking quantity at follow-up. We used a regression analysis of change in quantity of alcohol consumed per occasion with a difference score of the Readiness to Change Ladder (post minus baseline) and the IAT score entered as Step 1 and a product of the standardized values of the difference score for the Readiness to Change Ladder and IAT score entered as Step 2. The results (Table 2) indicate an interaction effect (*β* = .23, *p* = 0.03). Simple slope analyses indicated that higher readiness to change drinking was significantly associated with reductions in drinking among those who demonstrated weak automatic alcohol-approach associations on the IAT (−1 *SD*) (*t* = −2.9, *p* = 0.005). Again, the effect of this self-report measure of readiness to change was not associated with reductions in drinking among those who exhibited strong automatic alcohol-approach associations on the IAT (+1 *SD*) (*t* = 0.12, *p* = 0.91). These findings are illustrated in Figure 2.

**Table 2:** **Automatic appetitive responses to alcohol moderate the relation between readiness to change drinking and changes in alcohol consumed per occasion** (**N = 87**)

	**Variable**	***R***-***squared change***	***F***-***change***	***Beta***
***Change in quantity of alcohol consumed per occasion between baseline*** &***follow***-***up***				
Step 1				
	IAT Score	0.08	*F* (2, 83) = 3.63	0.21
	RTC Difference			−0.20
Step 2				
	IAT x RTC Difference	0.05	*F* (1, 82) = 4.84	0.23*

*Note*: Change in quantity of alcohol consumed per occasion = follow-up quantity/occasion – baseline quantity/occasion (larger values indicate increased consumption); RTC Difference = post-intervention Readiness to Change Ladder – baseline Readiness to Change Ladder (larger values indicate greater motivation to reduce drinking); IAT score (larger scores = stronger automatic appetitive responses toward alcohol).

*p < 0.05.

## Discussion

The current study examined whether automatic alcohol-approach associations would moderate the influence of a BMI on subsequent drinking quantity. The findings supported this hypothesis in that the BMI led to greater reductions in alcohol consumed per occasion for participants who had weak alcohol-approach (relative to alcohol-avoid) associations. The significance of determining moderators of BMIs is underscored by the finding that there were no main effects of this BMI on reductions in drinking quantity at follow-up. Further analyses indicated that the BMI increased motivation to reduce drinking and that the relation between motivation to change and reduction in drinking at follow-up was moderated by automatic alcohol-approach (relative to alcohol-avoid) associations. This measure of readiness to change only appeared to predict reductions in alcohol use among those who showed weaker automatic alcohol-approach associations. These findings suggest that although an intervention consisting of a discussion of negative alcohol-related consequences conducted in an empathic style can alter the perceived costs of drinking and thus increase motivation to reduce alcohol consumption, this may not be enough to effect change. Rather, translating motivation to reduce alcohol use into actual change may be particularly difficult for those who exhibit strong automatic alcohol-approach associations toward alcohol.

These results support the importance of assessing automatic processes in order to predict behavior. Dual-process models of the mind often suggest that the default mode is one in which automatic (nonvolitional) processes influence behavior and that controlled (volitional) processes are activated only in the case in which an individual has both the motivation and the resources to inhibit automatic responses [19]. Given evidence that (a) addictive behaviors represent the breakdown of the ability of controlled processes to influence substance use behavior [45], and (b) implicit measures may be better able than explicit measures to assess spontaneous (automatic) behavior [32,46-48], implicit measures hold potential for increasing our understanding of the psychological mechanisms underlying addiction. The current findings contribute to growing evidence that implicit measures predict alcohol use despite intentions to restrain consumption [24,32].

Several limitations should be taken into account when interpreting the results of the study. For example, the lack of a main effect of the BMI on follow-up drinking should be cautiously interpreted given the relatively short length of the intervention. Given that similarly short BMIs have also failed to find a main effect on reduced drinking [49], it is possible that longer interventions incorporating more motivational interviewing elements may be necessary for change to occur [10,12]. A second limitation consists of the study’s methodological elements that may have reduced the likelihood of detecting an intervention effect on drinking quantity, including the relatively small sample size, which would allow a power of approximately 0.60 to detect medium effect-size differences between groups and the relatively short follow-up period. A third limitation is that the sample, consisting of hazardous-drinking college students, limits the ability to make general statements about the findings. Future research with alcohol-dependent adults would contribute to the understanding of automatic processes as treatment moderators. A fourth limitation consists of the fact that the IAT creates a summary score that incorporates both approach and avoidance associations with alcohol. There is evidence that the IAT used in this study is more reflective of approach motivation toward alcohol rather than avoidance motivation away from alcohol [31], but future research would benefit from using implicit measures such as the single-category IAT [50] to examine the independent contributions of approach and avoidance associations in predicting treatment outcome.

Despite these limitations and a nonsignificant effect of the BMI on subsequent alcohol consumption, the findings have a number of potential clinical implications. First, they suggest that assessing automatic alcohol-approach associations may be useful in predicting which heavy drinkers will improve after a BMI and which may need a longer and more intense intervention. This sort of prescreening could improve the efficiency of using limited intervention resources.

Second, the results from the current study have potential implications for treatment development. BMIs and other common treatments may not be adequate for changing automatic processes related to alcohol. For example, a study that administered an expectancy challenge designed to reduce positive alcohol outcome expectancies found that although the intervention led to changes in an explicit measure of alcohol motivation, it did not lead to changes in an implicit measure and had negligible effects on drinking behavior [51]. Other research has similarly found that inducing a discrepancy between values and behavior may change explicit but not implicit measures of attitudes [52]. Such findings underscore the importance of considering how to directly address automatic processes in interventions. Initial studies suggest that strategies designed to change automatic associative and attentional biases may lead to reductions in problematic alcohol use [53,54]. Other approaches, such as developing automatic self-regulation of urges [55] or using mindfulness interventions [56,57] may help to decouple the relation between automatic alcohol-approach associations and drinking behavior. Development of BMI treatment may also benefit from attempts to examine which elements of the intervention prime appetitive motivation to consume alcohol, as the current study found that a BMI led to increased drinking for participants with strong automatic approach (relative to avoid) responses toward alcohol (as has been found in other research [58]).

Addictive behaviors are notoriously difficult to change. This is demonstrated in high relapse rates [59] and other indices of dyscontrol [45]. The current findings suggest that automatic alcohol-approach associations may contribute to the dyscontrolled nature of alcohol use disorders. The current study also suggests that increasing our understanding of the role of automatic processes in alcohol use will help to develop more efficacious treatments.

## Competing interests

The authors have no competing interests.

## Authors’ contributions

Both authors contributed to the design of the study, data analyses and the writing of the manuscript. Both authors read and approved the final manuscript.
